# Stable Carbon Isotope Composition of the Lipids in Natural *Ophiocordyceps sinensis* from Major Habitats in China and Its Substitutes

**DOI:** 10.3390/molecules22091567

**Published:** 2017-09-18

**Authors:** Lian-Xian Guo, Xiao-Ming Xu, Yue-Hui Hong, Yan Li, Jiang-Hai Wang

**Affiliations:** 1 Dongguan Key Laboratory of Environmental Medicine, School of Public Health, Guangdong Medical University, Dongguan 523808, China; glx525@163.com; 2 Guangdong Provincial Key Laboratory of Marine Resources and Coastal Engineering/South China Sea Bioresource Exploitation and Utilization Collaborative Innovation Center, School of Marine Sciences, Sun Yat-Sen University, Guangzhou 510006, China; xuxiaom8@mail.sysu.edu.cn (X.-M.X.); hongyh6@mail.sysu.edu.cn (Y.-H.H.)

**Keywords:** stable carbon isotope analysis, lipids, fatty acids, *Ophiocordyceps sinensis*, the Qinghai-Tibetan Plateau

## Abstract

*Ophiocordyceps sinensis* is one rare medicinal fungus produced in the Qinghai-Tibetan Plateau. Its quality and price varies hugely with different habitat, and its numerous substitutes have sprung up in functional food markets. This paper aims to discriminate the geographic origin of wild *O. sinensis* and its substitutes via element analyzer–isotope ratio mass spectrometry and gas chromatography–isotope ratio mass spectrometry. The δ^13^C values of major fatty acids in the lipids of *O. sinensis* are characterized unanimously by the variation relation C_18:0_ < C_18:2_ ≈ C_16:0_ < C_18:1_, while their fluctuation intervals are notably different between those of neutral and polar lipids. The comparative analysis of the δ^13^C ratios of major fatty acids in lipids of *O. sinensis* suggests that the δ^13^C patterns may be sensitive potential indicators to discriminate its geographical origin. The δ^13^C values of individual major fatty acids of lipids from the cultivated stromata of *Cordyceps militaris* (SCM), the fermented mycelia of *Hirsurella sinensis* (FM_H_) and *Paecilomyces epiali* (FM_P_) range from −31.2‰ to −29.7‰, −16.9‰ to −14.3‰, and −26.5‰ to −23.9‰, respectively. Their δ^13^C pattern of individual major fatty acids may be used as a potential indicator to discriminate the products of natural *O. sinensis* and its substitutes.

## 1. Introduction

*Ophiocordyceps sinensis*, popularly named as winter-worm-summer-grass (Dong Chong Xia Cao in Chinese), is one precious insect larva–fungus symbiote mainly occurred in the Qinghai-Tibetan Plateau [1,2]. The Latin term of *Ophiocordyceps sinensis* (*O. sinensis*) refers to the teleomorph stage of the larva–fungus complex, and has been renamed from *Cordyceps sinensis* since 2007 [3]. Its anamorph has been confirmed as *Hirsurella sinensis* based on the mainstream view [4]. In this paper, we use *O. sinensis* to refer to its teleomorph and anamorph according to the regulation that one fungus only has one Latin name [5].

For more than 2000 years in China, *O. sinensis* has been used as a rare functional food or traditional medicinal herb to promote health and treat diverse chronic diseases [6,7,8]. Recent studies have suggested that *O. sinensis* possesses various components with the wide biological activities [6,9]. The investigation of *O. sinensis* has attracted our great attention, and become a hot topic due to its specific pharmacological effects [10]. It is well known that the wild resource of *O. sinensis* is extremely scarce because of its obligate parasitism [11,12] and eco-geographical preference [1,2,13]. In the latest decade, the wild yield of *O. sinensis* has continuously decreased, while its retail prices have accordingly increased due to excessive excavation [14,15]. It is particularly noteworthy that the prices of wild *O. sinensis* evidently vary according to its quality that closely depends on different geographic origins [16].

To alleviate the contradiction between the supply and demand of *O. sinensis*, many studies have been accordingly carried out in various academic fields. Diverse studies have involved in its artificial cultivation [15], but the large-scale man-made development has still constrained by the key technique of the host larva being infected by *O. sinensis*. Owing to the existence of this issue, many investigators focused on studying its alternatives, such as artificial cultivation of other *Cordyceps* fungi isolated from the stroma of *O. sinensis* as well as their fermented products [17], or artificially synthesizing their secondary metabolites [8]. Although some progress has been made, these substitutes have not replaced wild *O. sinensis* due to the differences in their minor bioactive substances [18]. In terms of their chemical compositions, previous studies are mainly concentrated on water-soluble components. To further understand the lipids of wild *O. sinensis*, the fatty acid composition of neutral and polar lipids in wild *O. sinensis* has been measured from several high-altitude habitats [13]. Simultaneously, we further investigated the fatty acid composition of neutral and polar lipids in indoor-cultivated *O. sinensis* at a high-altitude laboratory in the Tibetan Plateau [19]. However, the so-called artificially cultured or synthesized products are not recommended in the recent situation of the unclear functional components in *O. sinensis*. It is consensus that the obvious function difference exists among artificial, semi-artificial and wild *O. sinensis*, which may result from the specific functional ingredients biosynthesized by the host *Thitarodes* larva and *O. sinensis* in extreme environments [13,19]. Recently, there is indeed the adulteration in the processed products of natural *O. sinensis* with its substitutes [20], which seriously hurts the consumers and also disturbs the markets. Therefore, it is of significance to establish some effective indicators for discriminating this kind of adulteration or even the geographical origin of wild *O. sinensis*.

Previously, empirical methods based on the morphology, color or odor have been widely applied to discriminate natural *O. sinensis* from its substitutes [21]. In recent decades, health foods derived from *O. sinensis* have been extremely popular [6], and the conventional methods have become powerless for identifying the adulteration of processed products of natural *O. sinensis* with its substitutes. Up to date, the HPLC fingerprint method seems to be most effective [22]. Stable carbon isotope technique has been widely used to study the circulation of materials in the biosphere due to its remarkable advantage in tracing the long-term diet of animals [23]. The δ^13^C values of organisms or foods have also been widely applied as sensitive markers for discriminating their geographical origins and food adulteration [24,25,26,27,28,29]. Our group had employed stable carbon isotope analysis to study the diet of the host *Thitarodes* larva in the habitat of *O. sinensis* and the fungus–larva relation in the formation of *O. sinensis*, revealing that the humic matters in habitat soils were also one alternative food of the host larva except conventional tender plant roots [30]; and the site near the head of the host larva was the initial target attacked by *O. sinensis* [31]. Although our group reported the fatty acid composition of polar and neutral lipids in *O. sinensis* [13,19], to our knowledge, no report has involved the stable carbon isotope composition of lipids in wild *O. sinensis* and its artificially cultivated substitutes. In the present study, stable carbon isotope composition of the dominant fatty acids in polar and neutral lipids is presented for natural *O. sinensis* in seven typical habitats in China and several *Cordyceps* substitutes. The result may be used to discuss the impact of environmental factors on lipid synthesis in the formation of *O. sinensis*, determine the geographical origin of wild *O. sinensis* and discriminate the products of natural *O. sinensis* and its substitutes.

## 2. Results and Discussion

### 2.1. δ^13^C Values of the Bulk Samples of O. sinensis

**Figure 1:**
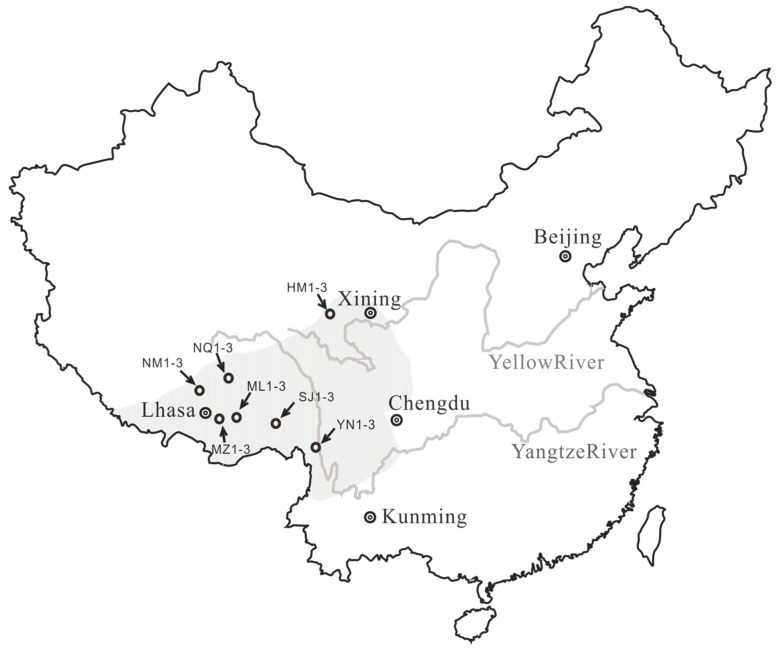
Schematic diagram illustrating the habitats of wild *Ophiocordyceps sinensis* in China (shadow area) and the sampling locations.

The δ^13^C values of the bulk samples of *O. sinensis* (Table 1) determined by elementary analyzer–isotope ratio mass spectrometer (EA–IRMS) are listed in Table 2. The results show that the δ^13^C values range from −27.5‰ to −25.3‰. The δ^13^C average values of *O. sinensis* in seven producing areas (Figure 1) were −26.2‰ (YN), −26.4‰ (HM), −25.5‰ (NQ), −26.6‰ (ML), −25.7‰ (NM), −26.9‰ (SJ), and −25.7‰ (MZ), respectively.

### 2.2. δ^13^C Values of Individual Major Fatty Acids of Neutral and Polar Lipids from O. sinensis and Its Substitutes

**Figure 2:**
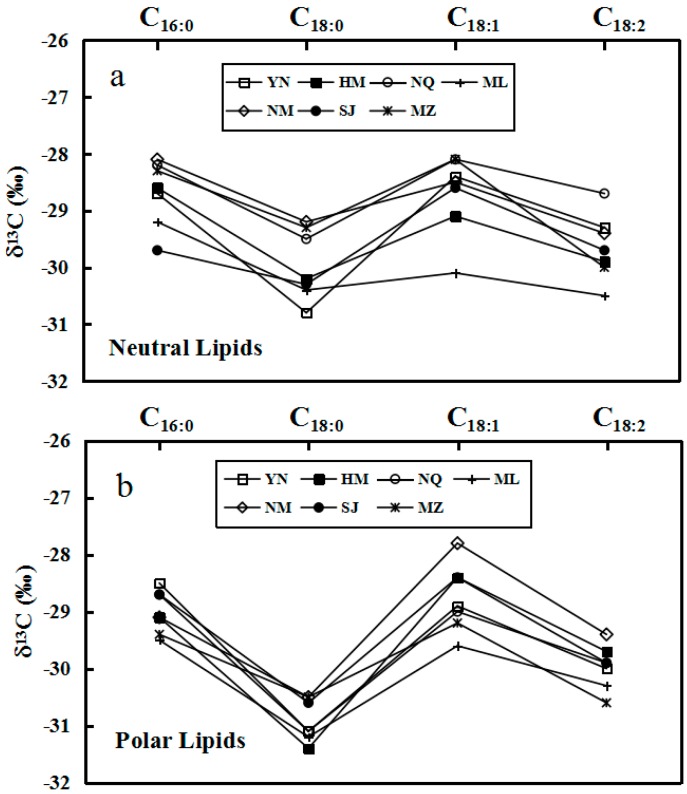
Variation of the stable carbon isotope ratios (δ^13^C) of fatty acids C_16:0_, C_18:0_, C_18:1_, and C_18:2_ in the neutral (**a**) and polar (**b**) lipids from wild *Ophiocordyceps sinensis.*

The δ^13^C values of individual major fatty acids of neutral and polar lipids from *O. sinensis* were determined by gas chromatography–isotope ratio mass spectrometer (GC–IRMS), as presented in Table 2. It can be seen in Table 2 that the δ^13^C values of major fatty acids C_16:0_, C_18:0_, C_18:1_ and C_18:2_ range from −31.4‰ to −27.8‰ for neutral lipids, and −31.9‰ to −27.5‰ for polar lipids, exhibiting the ^13^C depletion comparing with those of the bulk tissues. The δ^13^C values also disclosed a prominent fluctuation with the amplitudes of 3.6‰ for neutral lipids and 4.4‰ for polar lipids. The δ^13^C values of major fatty acids in neutral and polar lipids were characterized uniformly by the variation law C_18:0_ < C_18:2_ ≈ C_16:0_ < C_18:1_ (Table 2 and Figure 2). However, the fluctuation amplitudes were notably different between neutral and polar lipids. The δ^13^C values of C_16:0_ and C_18:0_ in neutral and polar lipids have the relationship of C16:0 > C_18:0_, and C_18:0_ has a pronounced ^13^C depletion of −0.4‰ to −2.9‰ in neutral lipids and −1.7‰ to −2.9‰ in polar lipids compared with C_16:0_. The δ^13^C values of C_18:0_ and C_18:1_ in neutral and polar lipids possess the relationship of C_18:0_ < C_18:1_, and C_18:1_ displays an evident ^13^C enrichment of +1.6‰ to +3.0‰ in neutral lipids and +2.2‰ to +3.6‰ in polar lipids. The δ^13^C values of C_18:1_ and C_18:2_ in neutral and polar lipids show the trend of C_18:1_ > C_18:2_, and C_18:2_ and exhibit the ^13^C depletion of −0.3‰ to −1.8‰ in neutral lipids and −0.2‰ to −2.1‰ in polar lipids.

The δ^13^C values of individual major fatty acids of neutral and polar lipids from the samples of SCM_N_, SCM_S_, FM_H_ and FM_P_ were presented in Table 3. The δ^13^C values of major fatty acids C_16:0_, C_18:0_, C_18:1_ and C_18:2_ in the samples of SCM_N_ and SCM_S_ range from −32.0‰ to −29.8‰ for neutral lipids and −31.2‰ to −29.7‰ for polar lipids, while the δ^13^C values of the four major fatty acids in the samples of FM_H_ are in the intervals of −16.8‰ to −14.6‰ for neutral lipids and −16.9‰ to −14.3‰ for polar lipids. For the samples of FM_P_, the δ^13^C values of the four major fatty acids are in the ranges of −26.5‰ to −24.2‰ for neutral lipids and −26.2‰ to −23.9‰ for polar lipids.

### 2.3. Stable Carbon Isotope Fractionation among Plants, Larvae and O. sinensis

Catabolism or synthetic metabolism of carbonaceous compounds may result in stable carbon isotope fractionation in organisms. Out of biological metabolisms, the fixation and release of CO_2_ are proven to be two notable processes that can induce stable carbon isotope fractionation. It is well known that the δ^13^C values have remarkable advantage in tracing carbon cycling in the food chain. Two stable carbon isotopes, i.e., ^12^C and ^13^C, are unevenly distributed in different compounds, which can be used to trace the chemical, physical and metabolic processes in carbon transfer [32]. For instance, C3 and C4 plants possess distinctly different δ^13^C values due to the isotope fractionation in the photosynthetic carbon fixation. Because heterotrophic organisms do not substantially alter the δ^13^C values of their foods [23], it is probable to assess the relative dependence of heterotrophic organisms on these isotopically distinct categories of primary producers.

**Figure 3:**
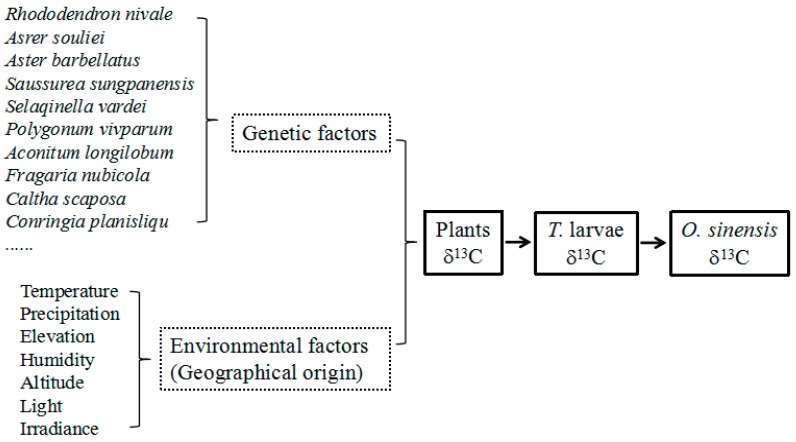
Impact factors on the δ^13^C values of *O. sinensis.*

**Figure 4:**
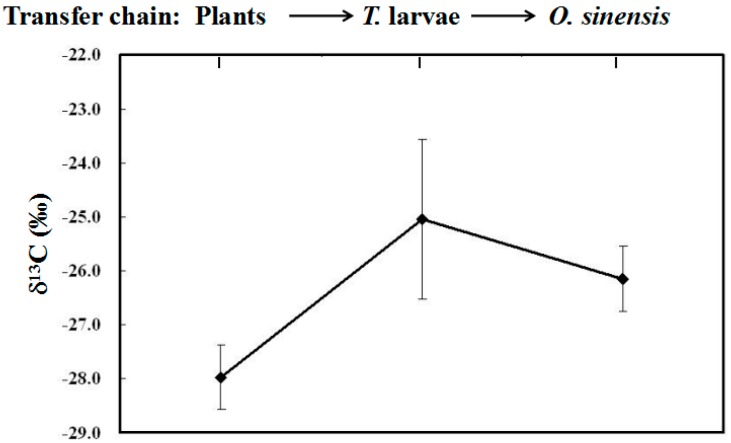
Variation of the stable carbon isotope ratios (δ^13^C) of the bulk samples of plants, larvae and *O. sinensis.*

The food chain among plants, larvae and *O. sinensis* in wild environments can be described as follows (Figure 3). The plants, whose tender roots are the favorite food for *Thitarodes* larvae, are at the bottom of the food chain *Thitarodes* larvae are the predator. *O. sinensis*, a fungus obligately parasitized on *Thitarodes* larvae, is the primary decomposer in the food chain. Theoretically, there is a significant correlation of the δ^13^C values among *O. sinensis*, *Thitarodes* larvae and plants. It can be seen in Figure 4 that there is an obvious variation of the δ^13^C values from plants to host larvae, and to *O. sinensis* based on our previous [30] and newly-obtained data. *Thitarodes* larva, which generally lives in deeper soils and mainly feeds on the tender roots of C3 plants [1], is the specific host of *O. sinensis*. Therefore, the δ^13^C values of *O. sinensis* clearly exhibit the specific δ^13^C ratios of C3 plants.

It has already reported that, for the decomposer, fungus, the average enrichment of δ^13^C values reached about 3.5‰ in comparison with its substrate [33]. However, the δ^13^C values of *O. sinensis* in this study are slightly less than those of the host larvae, which is in agreement with the result provided by Ruess et al. [34]. The above discrepancy among fungi suggests that stable carbon fractionation may also depend on different microbes. For *O. sinensis*, the increasing studies have proved that there is intrinsically the complexity among fungi and bacteria. The abundant diversity of microbes in the formation of *O. sinensis* may be one important reason resulting in its diverse bioactivities [35]. Thus, we consider that the combination of diverse microbial metabolisms in the host larva as one substrate may ultimately cause the specific stable carbon composition of *O. sinensis*.

### 2.4. Stable Carbon Isotope Fractionation in the Lipids of O. sinensis

The lipids in *O. sinensis* are derived, from either de novo synthesis or uptake of fatty acids from the host *Thitarodes* larva [36]. For de novo synthesis, C_16:0 in all studied samples_ of *O. sinensis* is considered to be the starting material for longer fatty acids and dehydro-fatty acids, and plays a central role in the biosynthesis of other fatty acids in *O. sinensis*, just as revealed in other fungi [37,38]. Theoretically, both elongation and desaturation of fatty acids in lipids from *O. sinensis* may result in the ^13^C depletion in comparison with the precursors in its parasitized larva, even though no carbon atoms are involved in desaturation since the addition of one neutron can considerably decrease the rate of a chemical reaction [39]. Our newly-obtained data have shown that the variation of δ^13^C values in the elongation from C_1__6__:0_ to C_18:__0_ and the second desaturation from C_18:__1_ to C_18:__2_ in *O. sinensis* are consistent with the above prognostication, while the evident ^13^C enrichment abnormally occurs in the first desaturation from C_1__8__:0_ to C_18:__1_ (Table 2 and Figure 2). This abnormity may be reasonably explained as follows. To adapt to the inclement environments, *O. sinensis* requires the plentiful polar lipids enriched in polyunsaturated fatty acids, in particular C_18:2_, for the larger proportion of cellular and subcellular biomembranes [19,40]. We had revealed that the absolute amount of total fatty acids and relative content of C_18:2_ in polar lipids from *O. sinensis* were evidently higher than those from its host larva, implying that *O. sinensis* assimilated polar lipids much more than those in the host larva and had transformed more C_18:1_ to C_18:2_ except a direct uptake of C_18:2_ from the parasitized larva. Thus, C_18:2_ in *O. sinensis* was originated both from the host larva and de novo synthesis. However, the host larva was unable to biosynthesize C_18:2_ [41,42], and had to assimilate C_18:2_ only from the tender roots of plants, especially *Polygonum macrophyllum* and *Polygonum viviparnm*, which were considered to be its favorite food with the δ^13^C value lower than that of the predator larva [30]. In the de novo synthesis of C_18:2_, the ^13^C-depleted C_18:1_ in *O. sinensis* was preferred to be desaturated. The formation of abundant C_18:2_ consequentially resulted in a remarkable ^13^C enrichment in C_18:1_ and a greater ^13^C depletion in C_18:2_ in *O. sinensis*.

The carbon isotope fractionation of major fatty acids in *O. sinensis* may reflect the complicated integration of biochemical processes and environmental factors [36], mainly be triggered by its cold-tolerance response. The above results have also demonstrated the importance of elucidating the metabolism and biosynthesis (carbon-chain elongation and desaturation) of fatty acids, which result in the discrimination of δ^13^C value observed in *O. sinensis*. This carbon isotope fractionation of individual compounds by fungi may also help to discriminate the distinct carbon sources of food webs and contaminants in environment studies [29,38].

### 2.5. Relationship between the δ^13^C Values of O. sinensis and Its Habitats

In our previous studies, the fatty acid composition of polar lipids was found to be a potential marker for distinguishing *O. sinensis* between Yunnan and the other habitats; and the fatty acid profile and ratio of C_18:1_/C_18:2_ in polar lipids were suggested to be two potential indicators for discriminating its geographical origin [13]. It is well known that the fatty acid composition of lipids and their δ^13^C values in organisms are closely related to their living environments, in particular temperature and humidity [43]. Furthermore, as mentioned above, the biochemical processes and environmental factors have a great impact on the δ^13^C pattern of major fatty acids. Thus, we here calculate the difference of the δ^13^C values of individual major fatty acids (Δ^13^C values) in neutral and polar lipids of *O. sinensis*, and discuss whether the Δ^13^C values may be a potential indicator for discriminating its geographical origin.

**Figure 5:**
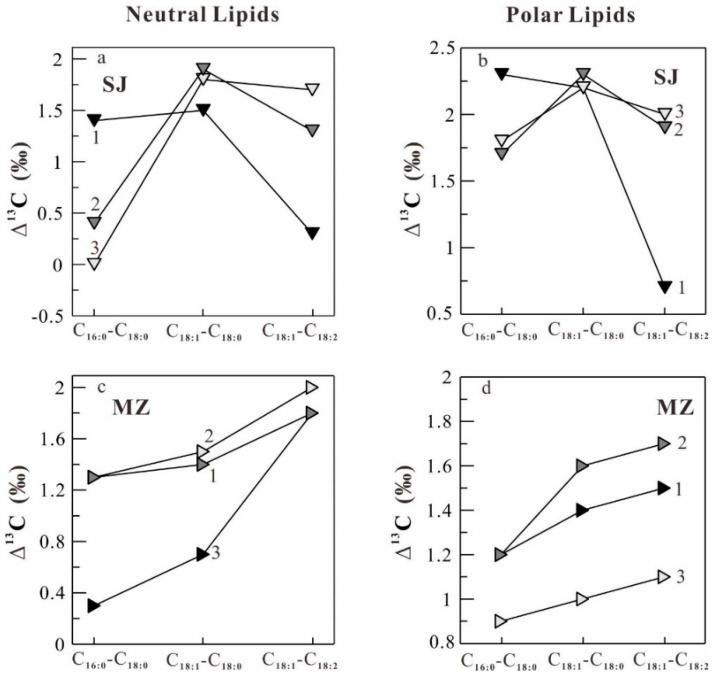
Difference of the δ^13^C values of major fatty acids (Δ^13^C) between the samples from Sejila Mountain (SJ, (**a**,**b**)) and Maizhokunggar (MZ, (**c**,**d**)). (**a**,**c**) and (**b**,**d**) refer to neutral and polar lipids, respectively.

**Figure 6:**
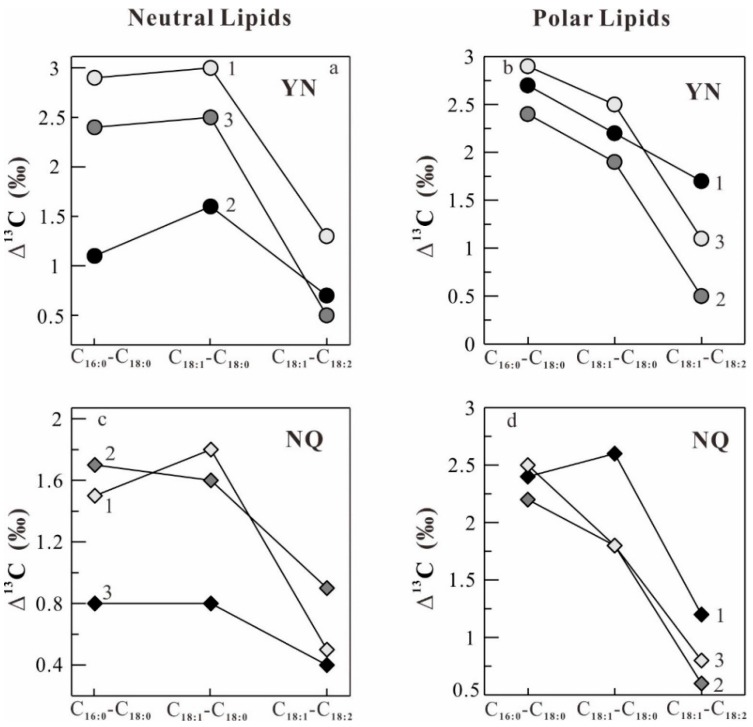
Difference of the δ^13^C values of major fatty acids (Δ^13^C) between the samples from Yunnan (YN, (**a**,**b**)) and Naqu (NQ, (**c**,**d**)). (**a**,**c**) and (**b**,**d**) refer to neutral and polar lipids, respectively.

**Figure 7:**
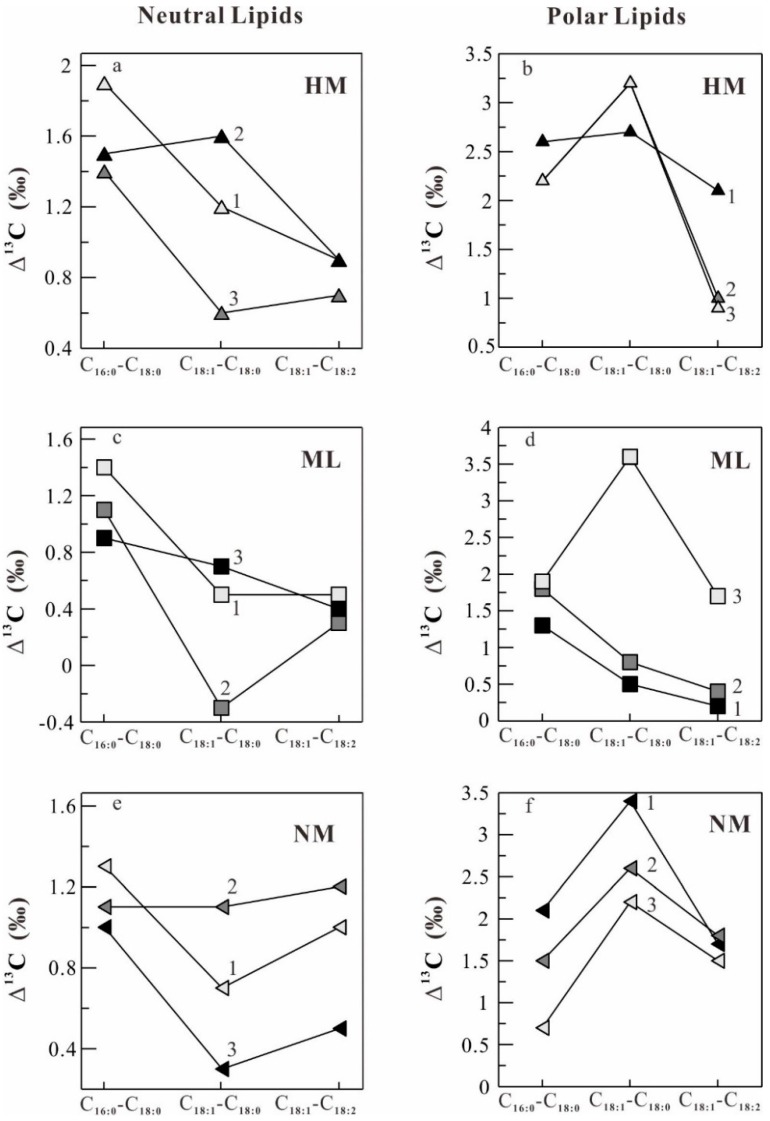
Difference of the δ^13^C values of major fatty acids (Δ^13^C) among the samples from Heimahe (HM, (**a**,**b**)), Mila Mountain (ML, (**c**,**d**)) and Nam Co (NM, (**e**,**f**)). (**a**,**c**,**e**) and (**b**,**d**,**f**) refer to neutral and polar lipids, respectively.

The δ^13^C patterns of difference of the δ^13^C values of individual major fatty acids (Δ^13^C values) in neutral and polar lipids in *O. sinensis* from seven different habitats (Figure 1) are illustrated in Figure 5, Figure 6 and Figure 7. It can be seen in Figure 5, Figure 6 and Figure 7 that the samples from Maizhokunggar (MZ) and Sejila Mountain (except SJ1) have the nearly similar δ^13^C patterns both in neutral and polar lipids, with the variation trends (Δ^13^C values) of C_18:1_–C_18:__2_ > C_18:1_–C_18:__0_ > C_1__6__:__0_–C_18:__0_ and C_18:1_–C_18:__0_ > C_18:1_–C_18:__2_ > C_1__6__:__0_–C_18:__0_, respectively (Figure 5). It is worth noting that the samples from Maizhokunggar (MZ) have the special δ^13^C patterns, with an increasing δ^13^C trend from C_1__6__:__0_–C_18:__0_ to C_18:1_–C_18:__0_, and to C_18:1_–C_18:__2_. The samples from Yunnan (YN) and Naqu (except NQ1) share the same δ^13^C patterns both in neutral and polar lipids, with the variation tendency of C_18:1_–C_18:__0_ > C_1__6__:__0_–C_18:__0_ > C_18:1_–C_18:__2_ for neutral lipids, while C_1__6__:__0_–C_18:__0_ > C_18:1_–C_18:__0_ > C_18:1_–C_18:__2_ for polar lipids (Figure 6). It can be seen from Figure 6 that the Δ^13^C values of major fatty acids in the samples from Yunnan (YN) are generally larger than those from Naqu (NQ); and there is an obvious difference in the δ^13^C patterns of neutral and polar lipids from both Yunnan and the other habitats (Figure 7). For instance, the samples from Mila Mountain (except ML3) possess the δ^13^C variation trend of C_1__6__:__0_–C_18:__0_ > C_18:1_–C_18:__2_ > C_18:1_–C_18:__0_ in neutral lipids and C_1__6__:__0_–C_18:__0_ > C_18:1_–C_18:__0_ > C_18:1_–C_18:__2_ in polar lipids; the samples from Heimahe (except HM2) have the δ^13^C profiles of C_1__6__:__0_–C_18:__0_ > C_18:1_–C_18:__0_ ≥ C_18:1_–C_18:__2_ in neutral lipids and C_18:1_–C_18:__0_ ≥ C_1__6__:__0_–C_18:__0_ > C_18:1_–C_18:__2_ in polar lipids; the samples from Nam Co (except NM2) have the δ ^13^C tendencies of C_1__6__:__0_–C_18:__0_ > C_18:1_–C_18:__2_ > C_18:1_–C_18:__0_ in neutral lipids and C_18:1_–C_18:__0_ > C_18:1_–C_18:__2_ ≥ C_1__6__:__0_–C_18:__0_ in polar lipids. The different δ^13^C patterns of major fatty acids in neutral and polar lipids of wild *O. sinensis* from different habitats may exhibit a close relation to their living environments.

It should be pointed out that there are a few outliers in our data, implying that the δ^13^C values of *O. sinensis* may be affected by complicated factors. Based on the principle that “you are what you eat” as well as those described above, the δ^13^C values of *O. sinensis* were ultimately related to those of the plants at the bottom of the food chain (Figure 3). Thus, except the genetic factors, the δ^13^C values of plants were influenced by environmental factors, such as temperature, precipitation, elevation, humidity, altitude, light and irradiance [43]; and are dominated by environmental factors in different habitats on the premise of the similar or same species of plants in habitats [24]. It can further be inferred that the δ^13^C value of *O. sinensis* should significantly correlate with environmental factors in diverse habitats if the host larva eats the same foods. However, the foods of the larvae are actually diverse and not limited to particular plants, even including humic substances in habitat soils in the period of food deprivations [30]. Consequently, the δ^13^C outliers of *O. sinensis* may be resulted from the omnivory of the host larvae and their complicated food web. Obviously, more δ^13^C data of major fatty acids are required to further confirm the coupling relation between the habitat (geographic origin) and δ^13^C value of wild *O. sinensis*.

### 2.6. Discrimination between O. sinensis and Its Substitutes

*O. sinensis*, as an insect–fungus symbiosis and the treasury of bioactive substances, has been approved as a valuable functional food with diverse pharmacological activities in oriental countries. However, owing to the particularity of its habitats, obligatory parasitism, and complexity of its life history, as well as the over-exploitation and ecological disruption caused by human beings, the natural *O. sinensis* resources have become increasingly scarce and endangered [8]. The retail price of *O. sinensis* (15,000 USD/kg for the medium quality) was correspondingly elevated largely in the recent decade [44]. Various processed products of natural *O. sinensis* or its so-called perfect substitutes are widely merchandised in a *Cordyceps* market dually driven by profits and demand [8]. Among the above-mentioned *Cordyceps* products, one kind of tablets made up of the superfine grinding powder of natural *O. sinensis* was the most expensive in the Chinese health food markets. The economic incentives urge manufacturers to produce the adulterated tablets by using the relatively cheap substitutes. Thus, it is necessary to study some indicators to discriminate the adulteration of *O. sinensis* products.

**Figure 8:**
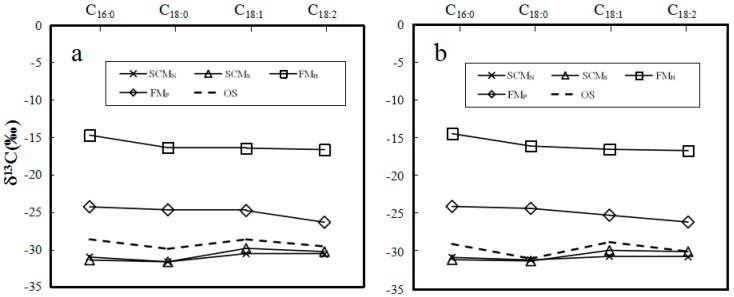
Variation of the δ^13^C values of major fatty acids C_16:0_, C_18:0_, C_18:1_, and C_18:2_ in the neutral (**a**) and polar (**b**) lipids from the common substitutes of *Ophiocordyceps sinensis.* SCM_N_ and SCM_P_, the cultivated stromata of *Cordyceps militaris*; FM_H_, the fermented mycelia of *Hirsurella sinensis*; FM_P_, the fermented mycelia of *Paecilomyces epiali*; OS, wild *Ophiocordyceps sinensis*.

Stable carbon isotope analysis has been used to monitor the quality and authenticate various foods [24,25,26,27,28,29]. In this study, the patterns of the average δ^13^C values of individual major fatty acids in the three *Cordyceps* substitutes (Figure 8) are evidently different from that of wild *O. sinensis* (as shown by the dashed line in Figure 8). Thus, the δ^13^C pattern may be one potential indicator to discriminate the adulteration of *O. sinensis* products. In other words, the δ^13^C values of authentic wild *O. sinensis* products are controlled by diverse factors (Figure 3), and are slightly lower than those of its host larva, but obviously higher than those of the favorite food plants of its host (Figure 4). Comparatively, the *Cordyceps* substitutes are generally produced via the large-scale cultivation or fermentation of the easily-cultured microbes in the formulated culture media at the low-elevation region. Therefore, both the genetic and environmental factors of the substitutes are completely different from wild *O. sinensis*.

It can be seen in Table 3 and Figure 8 that the lowest δ^13^C values of individual fatty acids occur in the SCM out of all studied samples, and range from −32.0‰ to −29.8‰ and −31.2‰ to −29.7‰ for natural and polar lipids, respectively. The stable carbon isotope composition suggests that the carbon source of the SCM is derived from C3 plants [45]. Similarly, the highest δ^13^C values of individual fatty acids appear in the FM_H_ among all the detected samples, and are at the intervals of −16.8‰ to −14.6‰ and −16.9‰ to −14.3‰ in natural and polar lipids, respectively. These δ^13^C values illustrate that the carbon source of the FM_H_ is originated from C4 plants. However, the δ^13^C values of major fatty acids in the FM_P_ are in the ranges of −26.5‰ to −24.2‰ and −26.2‰ to −23.9‰ in natural and polar lipids, respectively, indicating that its culture media may be a mixture of C3 and C4 plants. Except the discrepancy of the δ^13^C values among wild *O. sinensis* and its substitutes, the composition of their major fatty acids also has the specific variation trend. The δ^13^C patterns of FM_H_ and FM_P_ exhibit a slightly decreasing trend from C_16:0_ to C_18:2_; while those of SCM_N_ and SCM_S_ show a gently increasing trend (Figure 8). Both of them have the δ^13^C pattern different from that of wild *O. sinensis.* The uneven variation of these substitutes might be caused by the different δ^13^C value of carbon origin in the artificial medium. Obviously, the above δ^13^C patterns of individual major fatty acids may be potential signatures to discriminate wild *O. sinensis* from conventional *Cordyceps* substitutes.

## 3. Materials and Methods

### 3.1. Samples

Twenty-one samples of wild *O. sinensis* were collected from seven habitats in Yunnan, Qinghai and Tibet, China. Their masses range from 137.9 mg to 530.0 mg per single *O. sinensis*. The stromata and larval lengths of these samples were at the intervals of 2.10–4.76 cm and 2.57–4.95 cm, respectively. Their more detailed information and sampling sites are shown in Figure 1 and Table 1. The stroma samples of *Cordyceps militaris* (SCM) were purchased at two factories of artificially cultivating *Cordyceps militaris* in northern (SCM_N_ 1-3) and southern (SCM_S_ 1-3) China, respectively. The samples of FM_H_ 1-3 and FM_P_ 1-3 were purchased at the functional food market, and their strains were *Hirsurella sinensis* and *Paecilomyces epiali*. The samples of *O. sinensis*, *C. militaris* and fermented mycelia were dried and manually ground to fine-grained (less than 150 meshes) powders with a mortar and pestle.

### 3.2. δ^13^C Analysis of the Bulk Samples

The δ^13^C values of bulk samples were measured by element analyzer–isotope ratio mass spectrometry (EA-IRMS) with a CE EA1112 C/N/S analyzer (CE Instruments, Wigan, UK) interfaced with a Delta Plus XL mass spectrometer (Finnigan, Thermo Scientific, Waltham, MA, USA). In brief, ca. 2 mg of each powder sample was loaded into a clean tin capsule, which burned in an O_2_ atmosphere at 960 °C with helium as the carrier gas. One known δ^13^C value (−29.1‰, calibrated against the NBS-22 reference material with a δ^13^C value of −29.7‰) was used to calibrate a reference CO_2_ gas. One empty tin capsule was analyzed every batch of analyses to check the background. Instrument performance was routinely checked using a carbon black sample with the known δ^13^C value of −36.9‰. The corresponding standard deviation for each analysis was less than 0.3‰.

### 3.3. δ^13^C Analysis of Individual Major Fatty Acids in Neutral and Polar Lipids

The methods for preparation, extraction and trans-esterification of lipids followed the previous references [13,19]. Briefly, aliquots of the powder samples of each wild *O. sinensis* and about 500 mg *O. sinensis* substitutes were subjected to extract lipids with petroleum ether in an ultrasonic bath at room temperature for 5 min. The ultrasonic frequency was set at 40 kHz, and the ratio of the reagent to the material was 4:1. The suspension was filtered, and the residue was re-extracted with the same volumes of petroleum ether for additional three times. Extracts were combined and concentrated in a rotary evaporator, followed by reduction of the solvent to near dryness in a stream of N_2_. The yields were the extracts of the neutral lipids. Afterwards, the sample residues were re-extracted by using a solvent mixture of dichloromethane and methanol (1:1, *v*/*v*). This procedure was repeated at least four times. The products were then washed by distilled water to yield polar lipids. Thin-layer chromatography (TLC) was used to monitor each extraction procedure and check the purity of the products as visualized by using sulfuric acid as a spraying reagent.

Fatty acids in the neutral and polar lipids were further derivatized to be corresponding fatty acid methyl esters (FAMEs) before GC-IRMS analysis. In brief, a solution of neutral lipids in 2 mL anhydrous petroleum ether and 2 mL dichloromethane and methanol for polar lipids were prepared, respectively, before anhydrous tetrahydrofuran (1 mL) and sodium methoxide (5%, 1 mL) were added. After rigorous shaking and left to stand for ten minutes, the mixture was neutralized with 5% acetic acid (1 mL) and then washed with distilled water for three times to isolate fatty acid methyl esters (FAMEs).

The δ^13^C values of individual FAMEs were analyzed by using a 6890 gas chromatography (Agilent, Palo Alto, CA, USA) equipped with a split/splitless injector coupled to a combustion furnace to an isotope ratio mass spectrometer (IRMS, GV IsoPrime, Manchester, UK). The analytical conditions were identical to those used in our previous studies [46]. In brief, the injector was used in splitless mode at 290 °C; helium was the carrier gas at a constant flow rate of 1.0 mL per minute. A Varian WCOT fused silica column (50 m × 0.25 mm i.d. × 0.25 μm film thickness, CP-7419, Varian, Palo Alto, CA, USA) was used. The oven temperature was initially set at 100 °C (held for 2 min) and programmed to 190 °C at a heating rate of 6 °C/min (held for 5 min), and then programmed to 260 °C at a rate of 20 °C/min and held for 5 min.

The carbon isotope ratios were given as the δ-values (δ^13^C in ‰) relative to Vienna Pee Dee Belemnite standard (V-PDB), and they were corrected for the addition of carbon during the preparation of the FAMEs [46]. The average δ^13^C value of the methyl group was −32.5 ± 0.2‰ (six replicate analyses), and the δ^13^C values of the original fatty acids of samples were calculated according to the following equation:

δ^13^C_FA_ = [(*n* + 1) δ^13^C_FAME_ – δ^13^C_Methyl group_]/*n* (1)

where FA represents fatty acids, and *n* is the number of carbon atoms of the fatty acid. Each sample was analyzed thrice, and the standard deviations for FAMEs were at the interval of 0.15–0.27‰, showing the good reproducibility.

### 3.4. Statistical Analysis

The experimental data were analyzed by using the IBM SPSS Statistics (Version 20, Microsoft, Chicago, IL, USA). The δ^13^C value for each sample of *O. sinensis* was determined three times. Their standard deviations were <0.3‰, and are expressed as the mean values (Table 2 and Table 3).

## 4. Conclusions

The δ^13^C values of major fatty acids in the lipids of *O. sinensis* are characterized unanimously by the variation relation C_18:0_ < C_18:2_ ≈ C_16:0_ <C_18:1_, while their fluctuation intervals are notably different between the neutral and polar lipids, suggesting the mutual involvement of the complicated biochemical processes and environmental factors during the formation of *O. sinensis*. Stable carbon isotope fractionation in wild *O. sinensis* reflects the complicated influence from the biochemical processes and environmental factors. The δ^13^C profile of major fatty acids in the lipids may be a potential indicator to determine the geographical origin and quality of wild *O. sinensis* as well as authenticate the products of natural *O. sinensis*.

## Figures and Tables

**Table 1:** Description of twenty-one samples of *Ophiocordyceps sinensis* from Yunnan, Qinghai and Tibet, China.

Sample Nos.	Location	Longitude	Latitude	Weight (mg)	Larva Length (cm)	Stroma Length (cm)	Altitude (m)	Relative Humidity (%)
YN1	Deqin (Yunnan)	99°32′20 E	29°15′02 N	164.1	3.23	4.45	4250	71
YN2	Deqin (Yunnan)	99°32′20 E	29°15′02 N	530.0	4.95	2.13	4250	71
YN3	Deqin (Yunnan)	99°32′20 E	29°15′02 N	492.3	3.71	3.26	4250	71
HM1	Heimahe (Qinghai)	99°53′50 E	36°58′50 N	137.9	2.73	3.12	3310	49
HM2	Heimahe (Qinghai)	99°53′50 E	36°58′50 N	154.9	2.57	4.61	3310	49
HM3	Heimahe (Qinghai)	99°53′50 E	36°58′50 N	148.9	2.47	3.61	3310	49
NQ1	Naqu (Tibet)	93°02′20 E	31°55′02 N	434.2	4.08	2.13	4800	50
NQ2	Naqu (Tibet)	93°02′20 E	31°55′02 N	479.3	3.95	3.43	4800	50
NQ3	Naqu (Tibet)	93°02′20 E	31°55′02 N	449.3	3.85	3.13	4800	50
ML1	Mila Mountain (Tibet)	92°19′53 E	29°50′21 N	223.4	3.18	2.67	4825	46
ML2	Mila Mountain (Tibet)	92°19′53 E	29°50′21 N	235.1	3.21	2.95	4825	46
ML3	Mila Mountain (Tibet)	92°19′53 E	29°50′21 N	245.7	3.98	3.89	4825	46
NM1	Nam Co (Tibet)	90°16′50 E	30°35′18 N	510.0	4.34	3.39	4805	53
NM2	Nam Co (Tibet)	92°19′53 E	29°50′21 N	381.7	4.22	3.08	4805	53
NM3	Nam Co (Tibet)	92°19′53 E	29°50′21 N	397.1	4.32	3.28	4805	53
SJ1	Sejila Mountain (Tibet)	94°36′03 E	29°35′36 N	282.5	4.32	4.76	4241	79
SJ2	Sejila Mountain (Tibet)	94°36′03 E	29°35′36 N	189.5	3.95	3.46	4241	79
SJ3	Sejila Mountain (Tibet)	94°36′03 E	29°35′36 N	208.5	4.15	3.26	4241	79
MZ1	Maizhokunggar (Tibet)	91°48′03 E	29°50′36 N	322.3	3.93	3.32	4241	46
MZ2	Maizhokunggar (Tibet)	94°36′04 E	29°35′37 N	280.5	3.75	4.25	4241	46
MZ3	Maizhokunggar (Tibet)	94°36′05 E	29°35′38 N	222.3	3.25	3.45	4241	46

**Table 2:** δ^13^C values * (‰) of individual major fatty acids in neutral and polar lipids from *Ophiocordyceps sinensis.*

Sample Nos.	Bulk Sample	Neutral Lipids							Polar Lipids						
		C_16:0_	C_18:0_	C_18:1_	C_18:2_	C_16:0_–C_18:0_	C_18:1_–C_18:0_	C_18:1_–C_18:2_	C_16:0_	C_18:0_	C_18:1_	C_18:2_	C_16:0_–C_18:0_	C_18:1_–C_18:0_	C_18:1_–C_18:2_
YN1	−26.0	−28.5	−31.4	−28.4	−29.7	2.9	3.0	1.3	−28.2	−30.9	−28.7	−30.4	2.7	2.2	1.7
YN2	−25.5	−27.9	−30.3	−27.8	−28.3	2.4	2.5	0.5	−28.2	−30.6	−28.7	−29.2	2.4	1.9	0.5
YN3	−27.0	−29.6	−30.7	−29.1	−29.8	1.1	1.6	0.7	−29.0	−31.9	−29.4	−30.5	2.9	2.5	1.1
YN _AVR ± SD_	−26.2 ± 0.8	−28.7 ± 0.9	−30.8 ± 0.6	−28.4 ± 0.7	−29.3 ± 0.8	2.1	2.4	0.9	−28.5 ± 0.5	−31.1 ± 0.7	−28.9 ± 0.4	−30.0 ± 0.7	2.6	2.2	1.1
HM1	−26.2	−28.7	−30.2	−28.6	−29.5	1.5	1.6	0.9	−28.2	−30.8	−28.1	−30.2	2.6	2.7	2.1
HM2	−26.2	−28.7	−30.1	−29.5	−30.2	1.4	0.6	0.7	−29.7	−31.9	−28.7	−29.7	2.2	3.2	1.0
HM3	−26.8	−28.4	−30.3	−29.1	−30.0	1.9	1.2	0.9	−29.3	−31.5	−28.3	−29.2	2.2	3.2	0.9
HM _AVR ± SD_	−26.4 ± 0.4	−28.6 ± 0.2	−30.2 ± 0.1	−29.1 ± 0.5	−29.9 ± 0.4	1.6	1.1	0.8	−29.1 ± 0.8	−31.4 ± 0.6	−28.4 ± 0.3	−29.7 ± 0.5	2.3	3.0	1.3
NQ1	−25.8	−28.2	−29.7	−27.9	−28.4	1.5	1.8	0.5	−28.4	−30.8	−28.2	−29.4	2.4	2.6	1.2
NQ2	−25.6	−28.0	−29.7	−28.1	−29.0	1.7	1.6	0.9	−29.3	−31.5	−29.7	−30.3	2.2	1.8	0.6
NQ3	−25.3	−28.4	−29.2	−28.4	−28.8	0.8	0.8	0.4	−28.5	−31.0	−29.2	−30.0	2.5	1.8	0.8
NQ _AVR ± SD_	−25.6 ± 0.3	−28.2 ± 0.2	−29.5 ± 0.3	−28.1 ± 0.3	−28.7 ± 0.3	1.3	1.4	0.6	−28.7 ± 0.5	−31.1 ± 0.4	−29.0 ± 0.8	−29.9 ± 0.5	2.4	2.1	0.9
ML1	−26.7	−29.3	−30.7	−30.2	−30.7	1.4	0.5	0.5	−30.1	−31.4	−30.9	−31.1	1.3	0.5	0.2
ML2	−26.5	−29.1	−30.2	−30.5	−30.8	1.1	-0.3	0.3	−28.7	−30.5	−29.7	−30.1	1.8	0.8	0.4
ML3	−26.7	−29.3	−30.2	−29.5	−29.9	0.9	0.7	0.4	−29.8	−31.7	−28.1	−29.8	1.9	3.6	1.7
ML _AVR ± SD_	−26.6 ± 0.1	−29.2 ± 0.1	−30.4 ± 0.3	−30.1 ± 0.5	−30.5 ± 0.5	1.2	0.3	0.4	−29.5 ± 0.7	−31.2 ± 0.6	−29.6 ± 1.4	−30.3 ± 0.7	1.7	1.6	0.7
NM1	−25.8	−28.2	−29.5	−28.8	−29.8	1.3	0.7	1.0	−28.8	−30.9	−27.5	−29.2	2.1	3.4	1.7
NM2	−25.8	−28.2	−29.3	−28.2	−29.4	1.1	1.1	1.2	−29.0	−30.5	−27.9	−29.7	1.5	2.6	1.8
NM3	−25.5	−27.8	−28.8	−28.5	−29.0	1.0	0.3	0.5	−29.4	−30.1	−27.9	−29.4	0.7	2.2	1.5
NM _AVR ± SD_	−25.7 ± 0.2	−28.1 ± 0.2	−29.2 ± 0.4	−28.5 ± 0.3	−29.4 ± 0.4	1.1	0.7	0.9	−29.1 ± 0.3	−30.5 ± 0.4	−27.8 ± 0.2	−29.4 ± 0.3	1.4	2.7	1.6
SJ1	−26.2	−28.7	−30.1	−28.6	−28.9	1.4	1.5	0.3	−28.7	−31.0	−28.8	−29.5	2.3	2.2	0.7
SJ2	−27.5	−30.2	−30.6	−28.7	−30.0	0.4	1.9	1.3	−28.8	−30.5	−28.2	−30.1	1.7	2.3	1.9
SJ3	−27.0	−30.3	−30.3	−28.5	−30.2	0.0	1.8	1.7	−28.5	−30.3	−28.1	−30.1	1.8	2.2	2.0
SJ _AVR ± SD_	−26.9 ± 0.7	−29.7 ± 0.9	−30.3 ± 0.3	−28.6 ± 0.1	−29.7 ± 0.7	0.6	1.7	1.1	−28.7 ± 0.2	−30.6 ± 0.4	−28.4 ± 0.4	−29.9 ± 0.4	1.9	2.2	1.5
MZ1	−25.6	−28.0	−29.3	−27.9	−29.7	1.3	1.4	1.8	−29.3	−30.5	−29.1	−30.6	1.2	1.4	1.5
MZ2	−25.7	−28.2	−29.5	−28.0	−30.0	1.3	1.5	2.0	−29.5	−30.7	−29.1	−30.8	1.2	1.6	1.7
MZ3	−25.8	−28.8	−29.1	−28.4	−30.2	0.3	0.7	1.8	−29.5	−30.4	−29.4	−30.5	0.9	1.0	1.1
MZ _AVR ± SD_	−25.7 ± 0.1	−28.3 ± 0.4	−29.3 ± 0.2	−28.1 ± 0.3	−30.0 ± 0.3	1.0	1.2	1.9	−29.4 ± 0.1	−30.5 ± 0.2	−29.2 ± 0.2	−30.6 ± 0.2	1.1	1.3	1.4

* The δ^13^C values are the means of three determinations, and have their typical standard deviations (SD) of ≤0.25‰ (SD_C16:0_ ≤ 0.13‰; SD_C18:0_ ≤ 0.25‰; SD_C18:1_ ≤ 0.19‰; and SD_C18:2_ ≤ 0.21‰). AVR represents the average.

**Table 3:** δ^13^C values (‰) of individual major fatty acids in neutral and polar lipids from *O. sinensis* substitutes *.

Sample Nos. **	Neutral Lipids				Polar Lipids			
	C_16:0_	C_18:0_	C_18:1_	C_18:2_	C_16:0_	C_18:0_	C_18:1_	C_18:2_
SCM_N_ 1	−31.0	−31.5	−30.4	−30.4	−30.5	−30.8	−30.4	−30.4
SCM_N_ 2	−30.7	−31.6	−30.5	−30.6	−30.6	−30.9	−30.5	−30.6
SCM_N_ 3	−31.5	−32.0	−30.9	−31.1	−31.2	−31.6	−31.0	−31.1
SCM_N AVR ± SD_	−31.1 ± 0.4	−31.7 ± 0.3	−30.6 ± 0.3	−30.7 ± 0.4	−30.8 ± 0.4	−31.1 ± 0.4	−30.6 ± 0.4	−30.7 ± 0.4
SCM_S_ 1	−31.4	−31.7	−30.0	−30.3	−31.2	−31.2	−30.0	−30.1
SCM_S_ 2	−31.2	−31.4	−29.8	−30.5	−30.9	−31.1	−29.8	−30.0
SCM_S_ 3	−31.8	−31.9	−29.9	−30.2	−31.1	−31.4	−29.7	−29.9
SCM_S AVR ± SD_	−31.5 ± 0.3	−31.7 ± 0.3	−29.9 ± 0.1	−30.3 ± 0.2	−31.1 ± 0.2	−31.2 ± 0.2	−29.8 ± 0.2	−30.0 ± 0.1
FM_H_ 1	−14.7	−16.2	−16.4	−16.6	−14.5	−15.9	−16.4	−16.5
FM_H_ 2	−14.6	−16.4	−16.3	−16.6	−14.3	−16.0	−16.3	−16.6
FM_H_ 3	−14.9	−16.6	−16.7	−16.8	−14.5	−16.3	−16.8	−16.9
FM_H AVR ± SD_	−14.7 ± 0.2	−16.4 ± 0.2	−16.5 ± 0.2	−16.7 ± 0.1	−14.4 ± 0.1	−16.1 ± 0.2	−16.5 ± 0.3	−16.7 ± 0.2
FM_P_ 1	−24.5	−24.8	−25.0	−26.5	−24.2	−24.4	−25.3	−26.2
FM_P_ 2	−24.3	−24.8	−24.8	−26.3	−23.9	−24.2	−25.2	−26.0
FM_P_ 3	−24.2	−24.6	−24.7	−26.4	−24.0	−24.3	−25.1	−26.1
FM_P AVR ± SD_	−24.3 ± 0.2	−24.7 ± 0.1	−24.8 ± 0.2	−26.4 ± 0.1	−24.0 ± 0.2	−24.3 ± 0.1	−25.2 ± 0.1	−26.1 ± 0.1

* The δ^13^C values are the means of three determinations, and have their typical standard deviations (SD) of ≤0.25‰ (SD_C16:0_ ≤ 0.13‰; SD_C18:0_≤ 0.25‰; SD_C18:1_ ≤ 0.19‰; and SD_C18:2_ ≤ 0.21‰). AVR represents the average; ** SCM, the cultivated stromata of *Cordyceps militaris*; FM, the fermented mycelia.
